# How the emergence of the omicron variant may change people’s attitudes toward the COVID-19 pandemic

**DOI:** 10.3389/fpsyg.2022.922470

**Published:** 2022-07-29

**Authors:** Yong Yang

**Affiliations:** School of Public Health, University of Memphis, Memphis, TN, United States

**Keywords:** COVID-19 pandemic, omicron variant, negative influence on daily life, concerns of infection risk, prediction of the ending of the pandemic

## Abstract

**Background:**

This study aims to examine people’s attitudes toward the COVID-19 pandemic before and after the emergence of the omicron variant.

**Methods:**

Data were collected between November 15 and December 14, 2021, and three attitudes were included, namely, the negative influence on daily life, concerns of infection risk, and prediction of the ending of the pandemic.

**Results:**

The majority of people perceived that daily life was at least somewhat negatively influenced by the COVID-19 pandemic, and they worried at least once a week about infection risk. After the emergence of the omicron variant, the perceived negative influence and concern of infection risk decreased slightly while the prediction of ending increased significantly. People who were infected by COVID-19 perceived more negative influence and more concern of infection risk, but were more optimistic about the ending of the pandemic. People who did not get a vaccine perceived less negative influence and less concern of infection risk, but were more pessimistic about the ending of the pandemic. The attitudes varied significantly by individual and contextual characteristics.

**Conclusion:**

The emergence of omicron significantly increased people’s predicted ending time of the pandemic but did not change people’s perception of the pandemic’s negative influence on daily life and concern of infection risk.

## Introduction

The ongoing COVID-19 pandemic impacts most, if not all, perspectives of our life. Despite the rollout of the COVID-19 vaccine, toward the end of 2021, the new cases per day were above 600 thousand and 100 thousand, globally and in the United States alone, respectively. The recently emerged omicron SARS-CoV-2 variant sparked another global alarm. The omicron variant is a variant of the virus that causes COVID-19, first reported to the World Health Organization (WHO) on November 24, 2021 from South Africa. On November 26, 2021, the WHO classified it as a variant of concern (CDC, 2021). Having an unprecedentedly large number of mutations, the omicron variant may be more contagious than the original SARS-CoV-2 virus (Torjesen, 2021). Besides, for the omicron variant, we have limited knowledge of the severity of the disease and the effectiveness of prior infection, existing vaccines, and current treatment (WHO, 2021). Similar to the previous variants such as beta and delta, the emergence of the omicron variant is resulting in new waves of infection, although we are uncertain about the magnitude (Karim and Karim, 2021; Vaughan, 2021; Gao et al., 2022). After about 2 years of frustration with the pandemic, the emergence of the omicron variant may impact people’s attitude toward the COVID-19 pandemic, for example, perception of the disturbance to our life, concerns of infection risk, and confidence about the pandemic control. To the best of our knowledge, few studies examined people’s attitudes toward the COVID-19 pandemic, particularly the influence of the omicron variant. Such studies are important because these attitudes not only reflect people’s view of the disease’s contagiousness and severity but, more importantly, knowledge of their attitudes will contribute to the design and implementation of intervention measurements that could be tailored to certain groups.

The attitudes toward the pandemic may vary by individual’s characteristics, reflecting not only the joint influence of multiple determinants from biological, social, economic, cultural, historical, and other perspectives but also the disproportional impact of the pandemic on some vulnerable groups (Mein, 2020; Webb Hooper et al., 2020). For example, minorities and groups with low socioeconomic status may have less access to healthcare resources, and thus, they may be more concerned about infection risk. People who were infected before may be more influenced by the pandemic, but may be less worried about their infection. The effect of COVID-19 vaccination may be less straightforward: vaccine uptake may decrease people’s concern about their own infection risk, but at the same time, people who refuse to uptake the vaccine may be because they are less worried about the risk of infection. The attitudes may also be associated with contextual factors. People living in different neighborhood conditions (e.g., urban or rural with different poverty levels) may not only be impacted by the pandemic differently but also be exposed to different environmental changes through the pandemic. For example, evidence showed that neighborhoods with low poverty not only had more health-promoting conditions before the pandemic but also tended to have more positive changes during the pandemic (Yang and Xiang, 2021). Also, rural residents were less concerned about the pandemic and less supportive of the government’s pandemic control measures (Chauhan et al., 2021). To a larger spatial extension, various social norms and cultures may exist at both local and regional levels. In the United States, people’s attitudes toward a vaccine and the control measures were largely different in different states. For example, in early December 2021, the ratio of adults who have at least one shot of the COVID-19 vaccine was 50% in Idaho compared with 88% in New Hampshire. Thus, understanding people’s attitudes toward the pandemic and the influence of the omicron variant will also contribute to addressing the disparities of the pandemic’s adverse effects.

This study aims to examine people’s attitudes toward the COVID-19 pandemic before and after the emergence of the omicron variant with three measures, namely, the negative influence on daily life, concerns of infection risk, and prediction of the ending of the pandemic. It should be noted that the end of the COVID-19 pandemic does not mean there are no COVID-19 cases anymore; rather, the pandemic may change to some endemics similar to influenza, maybe with a seasonable pattern or maybe sporadic in some regions.

## Methods

Respondents were recruited from Amazon Mechanical Turk (MTurk) (Keith et al., 2017), an online crowdsourcing platform. The survey was described as “a study aimed to understand how the coronavirus (COVID-19) outbreak has affected Americans’ daily life and health.” We collected participants’ demographics, if infected by COVID-19 or not, the status of the COVID-19 vaccination, general physical health and mental health, and residential zip code. Data were collected between 15 November and December 14, 2021. The analysis was conducted in January 2022. The survey took about 2 min, and each participant was compensated $0.20. The Institutional Review Board at the University of Memphis approved this study.

For the influence of the pandemic on daily life, we asked “During the past 30 days, to what extent did the pandemic negatively influence your daily life?,” and respondents selected among five Likert scales ranged from “a great deal,” “much,” “somewhat,” “little,” to “never.” For the concern of COVID-19 infection, we asked “During the past 30 days, how often have you worried about the possibility of getting a COVID-19 infection,” and respondents selected among five Likert scales, namely, every day, a few days in a week, about once a week, seldom or less than once a week, and never. For the ending time of the pandemic, we asked “In your estimation, when will the COVID-19 pandemic end in the United States? by “end,” we mean although there are still new infections emerging, these cases are largely under the control, and the spread of coronavirus looks similar to seasonal influenza,” and respondents selected among seven options from “ < 3 months,” “ ≥ 3 and < 6 months,” “ ≥ 6 and < 9 months,” “ ≥ 9 and < 12 months,” “ ≥ 1 and < 1.5 years,” “ ≥ 1.5 and < 2 years,” until “at least 2 years.”

Using participants’ residential zip codes, we extracted three variables, namely, urbanicity (urban, suburban, rural) from the Rural-Urban Commuting Area Codes (U.S. Department of Agriculture, 2020), neighborhood poverty (i.e., the percent of families living below the poverty threshold) from the 2010 United States Census data, and the ratio of COVID-19 vaccination in each US state (i.e., the percent of people receiving at least one shot of COVID-19 vaccines) (USA Facts, 2021).

In this study, we used the day of November 26, 2021, when the omicron variant was classified as a variant of concern by the WHO (CDC, 2021), to separate participants as those who finished the survey before and after the emergence of the omicron variant. Weights were added to ensure the demographics of participants both before and after the omicron variant match with the United States general population using the 2020 Census considering gender, age, race/ethnicity, educational attainment, and household income. First, the three attitude outcomes were summarized both before and after the emergence of the omicron variant, with and without weights. Second, the mean value of each outcome was computed, stratified by the status of COVID-19 infection and vaccination, and before and after the emergence of the omicron variant. For the negative influence on daily life and concern of infection risk, the five Likert scales were coded into values from 1 to 5, with a higher value indicating a higher level of influence or concern. Similarly, the answer to the ending time of the pandemic was coded into values from 1 to 7, with a larger value indicating a long duration before the ending of the pandemic. Third, linear regressions were conducted to assess the association between the emergence of the omicron variant, the status of infection, and vaccination, with each of the three attitudes’ outcomes, adjusted by individual demographics and contextual factors.

## Results

As shown in Table 1, the whole sample included 3,239 participants, with 1,867 and 1,372 participants who finished the survey before and after the emergence of the omicron variant, respectively. The younger (18–24 years) and older adults (65 years and above), and those with low educational attainment were underrepresented in the sample. After weighting by demographics, the participants before and after the emergence of the omicron variant were roughly similar and matched the United States general population. The majority of participants lived in urban areas, in neighborhoods with moderate poverty, and in good and above physical and mental health status. About one-third of the participants were infected by COVID-19 and about 78% of them got the COVID-19 vaccine (at least one dose).

**TABLE 1:** Characteristics of participants.

Category	Item	Whole sample (*N* = 3220), unweighted	Before Omicron variant (*N* = 1862), weighted	After Omicron variant (*N* = 1358), weighted
Gender	Male	46.4	49.2	49.2
	Female	53.0	50.8	50.8
Age, in years	18–24	7.8	8.5	8.5
	25–34	40.7	17.0	17.0
	35–64	48.1	47.1	47.1
	65 and above	3.5	27.4	27.4
Race/ethnicity	White	65.4	60.3	60.3
	Black	10.7	13.4	13.4
	Asian	5.4	5.9	5.9
	Hispanic	16.6	18.5	18.5
	Other/mixed race	1.9	1.9	1.9
Educational attainment	High school or less	9.5	28.1	28.1
	Less than bachelor and more than high school	23.5	35.9	35.9
	Bachelor or higher	67.0	36	36
Household income	Less than $24,999	16.3	18.1	18.1
	$25,000–$49,999	27.3	20.3	20.3
	$50,000–$74,999	25.7	17.4	17.4
	$75,000–$99,999	16.4	12.8	12.8
	$100,000 or more	14.3	31.4	31.4
* Urbanization level	Urban	80.2	79	74.6
	Suburban	14.5	15.8	20.5
	Rural	5.3	5.2	5.0
Neighborhood poverty level	Low, ≤ 5%	10.4	8.0	12.9
	Moderate, > 5% - ≤20%	67.1	72.8	71.0
	High, > 20%	22.5	19.2	16.1
Physical health	Excellent	16.3	16.3	19.3
	Very good	34.8	32.1	31.6
	Good	35.8	37.8	30.8
	Fair	11.0	11.4	16.0
	Poor	2.1	2.4	2.2
Mental health	Excellent	13.0	18.6	15.2
	Very good	26.5	26.2	27.9
	Good	31.6	33.7	30.5
	Fair	21.2	15.1	20.2
	Poor	7.8	6.4	6.3
COVID-19 infection	Infected	33	31.2	33.0
	Has not been infected	67	68.8	67.0
COVID-19 vaccination	Yes	78.8	78.9	76.9
	No, but will do	8.6	7.6	6.7
	No, and will not do	12.6	13.5	16.4

The day of 26 November 2021 was used to separate participants as those who finished the survey before and after the emergence of the omicron variant. *The urbanization level was classified using the rural-urban commuting area (RUCA) codes (U.S. Department of Agriculture, 2020) from the home address’ zip codes, as urban (RUCA code 1), suburban (RUCA codes 2–6), and rural (RUCA codes 7–10).

As Table 2 shows, with weights, 66% of participants perceived that their daily lives were at least somewhat negatively influenced by the COVID-19 pandemic before the omicron variant and the percentage decreased to 60% afterward, and the percent of those who worried at least once a week for the risk of infection was 54 and 50% before and after the omicron variant, respectively. Before the emergence of the omicron variant, about 57% of participants believed that the pandemic will end within a year and the percentage decreased to 50% after the omicron variant. The percent of those who believed the pandemic will not end within 2 years was 25% before the omicron variant and increased to 33% afterward.

**TABLE 2:** The weighted distributions (%) of three perception outcomes, before and after the emergence of the omicron variant.

Before or after the emergence of the omicron variant		Before, *N* = 1862	After, *N* = 1358
Negative influence	A great deal	14.4	15.6
	Much	16.5	15.9
	Somewhat	35.1	28.4
	Little	23.6	31.7
	Never	10.5	8.4
Concern of infection	Everyday	18.9	16.1
	A few days in a week	20.0	18.4
	About once a week	14.7	15.3
	Seldom or less than once a week	29.2	33.0
	Never	17.3	17.3
Prediction of ending	< 3 months	10.4	8.5
	≥ 3 and < 6 months	14.2	11.0
	≥ 6 and < 9 months	14.7	12.0
	≥ 9 and < 12 months	17.7	18.9
	≥ 1 and < 1.5 years	10.4	10.9
	≥ 1.5 and < 2 years	7.8	6.4
	≥ 2 years	24.8	32.5

As the subgroups by the status of COVID-19 infection and vaccination (see Table 3), compared with those who had not been infected, those who were infected by COVID-19 before perceived more negative influence and more concern of infection risk, but were more optimistic about the ending of the pandemic in general. People who got the vaccine had roughly similar outcomes compared with those who didn’t get the vaccine but will get a vaccine. However, compared with those who got a vaccine, people who will not get a vaccine perceived much less negative influence and much less concern of infection risk, but were more pessimistic about the ending of the pandemic. In the comparison before and after the emergence of the omicron variant, most subgroups were consistent, that is, a slight or moderate increase in the perceived negative influence, a moderate decrease in the concern of infection risk, and the prediction of ending increased more significantly. Two subgroups are exceptional. First, the concern of infection risk was increased after the omicron variant among only one group: those who had not but will take a vaccine. Second, the prediction of ending was decreased after the omicron variant among only one group: those who will not take a vaccine.

**TABLE 3:** The weighted percentages of participants who perceived their lives were negatively influenced by the COVID-19 pandemic, those who worried about COVID-19 infection frequently, and those who believed that there would be at least another year before the end of the pandemic, stratified by the status of COVID-19 infection and vaccine uptake.

		^1^Negatively influenced		^2^Worried about infection		^3^Pessimistic about the pandemic ending	
Before/after the emergence of the omicron variant		Before	After	Before	After	Before	After
COVID-19 infection	No	27.3	27.6	33.8	30.5	46.4	55.3
	Yes	38.7	39.5	49.8	42.3	35.4	38.4
Vaccine uptake	Yes	32.3	32.8	44	38.7	41.7	48.9
	No but will	39.8	48.2	26.5	48.8	29.2	48.2
	No and will not	17.1	18.5	15.2	8.2	58.3	54.1

^1^The percentage of participants who perceived the extent of the pandemic negatively influencing their daily life was either a great deal or much.

^2^The percentage of participants who worried about the possibility of getting a COVID-19 infection either everyday or a few days in a week.

^3^The percentage includes participants who estimated that it would take at least another year before the COVID-19 pandemic would end.

As Table 4 shows, with the adjustment of several individuals and contextual variables, the emergence of the omicron variant was associated with a significant increase in the prediction of pandemic ending time and no change in the perceived negative influence and concern of infection risk. Hispanics, people with better physical health or worse mental health, those infected by COVID-19, and those who got COVID-19 vaccination tended to perceive a higher level of the negative influence of the COVID-19 pandemic on their daily life than their counterparts. Females, middle-aged adults, Hispanics, people with worse physical health or better mental health, those infected by COVID-19, those who got COVID-19 vaccination, and people living in states with a higher percent of vaccination tended to worry more frequently about COVID-19 infection compared with their counterparts. Females, younger adults, people with lower educational attainment or higher household income, those who had not been infected by COVID-19, and people living in suburban areas tended to be more pessimistic about the ending of the pandemic.

**TABLE 4:** Results of logistic regression models for the associations between variables with the three perception outcomes.

		^1^Negatively influenced	^2^Worried about infection	^3^Pessimistic about the pandemic ending
The emergence of Omicron variant	Before (ref)	0	0	0
	After	1.02 (0.86,1.21)	0.89 (0.74,1.06)	1.25 (1.06,1.47)**
Gender	Male (ref)	0	0	0
	Female	0.92 (0.77,1.1)	1.44 (1.2,1.73)**	1.73 (1.46,2.05)**
Age, in years	18–24 (ref)	0	0	0
	25–34	0.65 (0.46,0.93)*	1.36 (0.93,2)	0.65 (0.46,0.92)*
	35–64	0.82 (0.6,1.12)	1.72 (1.22,2.43)**	**0.67 (0.49,0.92)** *
	65 and above	0.41 (0.28,0.59)**	0.57 (0.38,0.85)**	0.39 (0.27,0.56)**
Race/ethnicity	White (ref)	0	0	0
	Black	1.13 (0.86,1.49)	0.62 (0.46,0.83)**	1.13 (0.88,1.47)
	Asian	0.76 (0.52,1.12)	1.03 (0.72,1.49)	0.97 (0.68,1.39)
	Hispanic	1.47 (1.16,1.87)**	1.91 (1.5,2.43)**	0.46 (0.36,0.59)**
	Others	0.54 (0.27,1.09)	2.44 (1.31,4.52)**	0.41 (0.21,0.78)**
^4^ Educational attainment		0.94 (0.84,1.07)	1.1 (0.97,1.24)	0.7 (0.62,0.79)**
^5^ Household income		0.99 (0.93,1.06)	0.85 (0.8,0.91)**	1.13 (1.06,1.2)**
^6^ Physical health		0.74 (0.66,0.82)**	1.13 (1.01,1.26)*	1.13 (1.02,1.25)*
^6^ Mental health		1.29 (1.17,1.42)**	0.88 (0.79,0.97)**	1.1 (1.01,1.21)*
COVID-19 infection	No (ref)	0	0	0
	Yes	1.35 (1.11,1.64)**	1.61 (1.32,1.96)**	0.68 (0.56,0.82)**
COVID-19 vaccination	Yes (ref)	0	0	0
	No, but will	1.22 (0.88,1.67)	0.52 (0.37,0.73)**	0.82 (0.59,1.15)
	No, and will not	0.46 (0.35,0.62)**	0.18 (0.12,0.25)**	1.1 (0.87,1.4)
^7^Urbanization level	Urban (ref)	0	0	0
	Suburban	0.82 (0.65,1.04)	0.62 (0.48,0.79)**	1.36 (1.1,1.69)**
	Rural	0.72 (0.46,1.11)	0.69 (0.45,1.07)	1.47 (1,2.16)
^8^Neighborhood poverty level		1.05 (0.89,1.24)	1.1 (0.93,1.31)	0.88 (0.75,1.04)
^9^State vaccination level		1.04 (0.93,1.15)	1.13 (1.02,1.25)*	1.1 (1,1.21)

Bold face indicates statistical significance, with * for *p* < 0.05 and ** for *p* < 0.01.

^1^Negatively influenced were participants who perceived that the extent of the pandemic negatively influencing their daily life was either a great deal or much.

^2^Worried about infection were participants who worried about the possibility of getting a COVID-19 infection either everyday or a few days in a week.

^3^Pessimistic about the pandemic ending were participants who estimated that it would take at least another year before the COVID-19 pandemic would end.

^4^Education attainment is coded into three levels, namely, 1 for high school and below; 2 for above high school and below bachelor; and 3 for bachelor and above.

^5^Household income is coded into five levels, namely, 1 for less than $24,999; 2 for $25,000–$49,999; 3 for $50,000–$74,999; 4 for $75,000–$99,999; and 5 for $100,000 or more.

^6^Both physical health and mental health are coded into five levels, namely, 1 for excellent; 2 for very good; 3 for good; 4 for fair; and 5 for poor.

^7^Urbanization level is categorized using the rural–urban commuting area (RUCA) codes (U.S. Department of Agriculture, 2020) from the home address’ zip codes as three levels, namely, (1) urban (RUCA code 1), (2) suburban (codes 2–6), and (3) rural (codes 7–10).

^8^Neighborhood poverty level is coded into three levels, namely, 1 for ≤ 5% of residents who were below the poverty line, 2 for > 5% and ≤ 20%, and 3 for > 20%.

^9^State vaccination level is coded into three levels, namely, 1 for < 65% of residents got at least one shot of COVID-19 vaccine, 2 for ≥ 65 and < 75%, and 3 for ≥ 75% until December 5, 2021 (USA Facts, 2021).

## Discussions

The majority of people perceived that their daily life was at least somewhat negatively influenced by the COVID-19 pandemic, and they worry at least once a week about the risk of infection. The emergence of omicron variant significantly increased people’s predicted ending time of the pandemic but did not change much of people’s perception of the pandemic’s negative influence on their daily life and their worry about the risk of infection. This may be explained by the nature of the omicron variant (CDC, 2022; Wang et al., 2022): compared with previous variants such as beta and delta, omicron has less severe symptoms, and thus, people’s worry about infection may not increase. At the same time, the omicron variant exhibits greater infectivity and thus may result in a new wave and prolong the pandemic.

Compared with those who had not been infected by COVID-19, infected people were more influenced by and more worried about, but were more optimistic about the ending of the pandemic. Infected people’s relatively higher optimism may be due to their overcoming of the COVID-19 (at least these participants survived and recovered largely, if not completely, from the disease). It may be not surprising to find that compared with those who got a COVID-19 vaccine, people who will not get a vaccine perceived less negative influence and much less worry about the pandemic because the perceived risk of disease is associated with the willingness of vaccination (Baumgaertner et al., 2020; Karlsson et al., 2021). Interestingly, for the group who had not but will take a vaccine, their worry about infection increased after the emergence of the omicron variant. This group may be not able or reluctant to get a vaccine for a while due to various reasons, and the highly contagious omicron variant may change their perception of the risk more compared with those who got a vaccine and those who will not get a vaccine. Also, for the group who will not get a vaccine, their prediction of the pandemic ending decreased after the omicron variant. One possible explanation is that for this group who were unlikely to believe the effectiveness of the COVID-19 vaccine (Karlsson et al., 2021), the highly contagious omicron variant may speed up the natural immunity process, thus ending the pandemic earlier.

Our results confirmed that attitudes toward pandemics varied by some individual and contextual characteristics. For example, compared with male participants, female participants perceived a higher level of negative influence, more concerns about infection, and more pessimism about the ending of the pandemic. This is consistent with some evidence that men tend to be more optimistic than women for various issues (Jacobsen et al., 2014; Bjuggren and Elert, 2019) and recent findings that women were disproportionately impacted (e.g., the burden of child care and more likely to lose employment) (Skinner et al., 2021; Zamarro and Prados, 2021) and tended to perceive and expect the COVID-19 pandemic more negatively than men (Dolinski et al., 2020; Alsharawy et al., 2021). Our results showed that middle-aged adults were more worried about the infection which may be due to the fact the middle-aged adults tended to take multiple roles and they may be obligated to interact more frequently with the outside which may increase their exposure to the COVID-19 virus. Although we did not find significant differences in the negative influence on daily life by race/ethnicity (except Hispanic), education, and household income, our results indicated that higher household income was associated with less concern of infection risk, higher educational attainment was associated with being more optimistic for the ending of the pandemic, and worse mental health was associated with more negative influence by the pandemic.

As contextual characteristics, we did not find large differences in the negative influence and concern of infection risk across groups living in urban, suburban, and rural areas, although people who lived in suburban and rural areas were more pessimistic about the ending of the pandemic. Also contrary to our expectations, the neighborhood poverty level was not associated with negative influence although a higher neighborhood poverty level was associated with a higher concern of the infection risk. We found that living in states with a higher ratio of vaccination was associated with more concern of infection risk. A possible explanation is that people’s higher concern of infection risk leads to a higher ratio of vaccination. Explaining each variation is beyond the aim of this study. For example, our results indicated that older adults were more optimistic about the ending of the pandemic, and Hispanics were more influenced by and more worried about, but were more optimistic about the ending of the pandemic. Further study is warranted for these interesting results.

One limitation of this study is that the sample is not a United States representative sample; although we added weights to match the sample with United States general population for major demographics, the online survey itself may exclude people who are illiterate or do not have access to the Internet. Second, taking advantage of a cross-sectional dataset that was collected between November and December 2021, we compared the attitudes toward the pandemic before and after the emergence of the omicron variant. Our findings may only reflect the immediate change of attitude, and more studies are needed to examine the influence of the omicron variant for a relatively long term, and longitudinal data will be ideal. Overall, our findings will help to design pandemic control and measurements, both mitigating the pandemic’s adverse effects in general and reducing the disparities for certain groups.

Our findings may contribute to intervention measurements. For example, identification of the groups who were disproportionally negatively impacted by the pandemic such as females and younger adults may help to design tailored interventions to mitigate the pandemic’s adverse effects. Also, insights into people’s concerns about infection risk could be leveraged to increase vaccination among some groups. For example, the worry of infection increased significantly after the emergence of the omicron variant among participants who had not but will take a vaccine. This indicates that the emergence of a new variant could be a unique opportunity to promote COVID-19 vaccination.

## Data availability statement

The raw data supporting the conclusions of this article will be made available by the authors, without undue reservation.

## Ethics statement

The studies involving human participants were reviewed and approved by the University of Memphis. The patients/participants provided their written informed consent to participate in this study.

## Author contributions

YY conceived the study, collected related data, conducted analyses, and drafted the manuscript.
